# Uncooled low-noise optomechanical sensors on thin-film lithium niobate

**DOI:** 10.1038/s41377-026-02506-z

**Published:** 2026-10-20

**Authors:** Yue Yu, Ran Yin, Ian Anderson, Yinan Wang, Jack Kramer, Ian Christen, Chun-Ho Lee, Xinyi Ren, Zaijun Chen, Michelle L. Povinelli, Daniel Wasserman, Ruochen Lu, Mengjie Yu

**Affiliations:** 1 Department of Electrical Engineering and Computer Sciences, University of California, Berkeley, Berkeley, CA 94720 USA; 2 Ming Hsieh Department of Electrical and Computer Engineering, University of Southern California, Los Angeles, CA 90089 USA; 3 Materials Sciences Division, Lawrence Berkeley National Laboratory, Berkeley, CA USA; 4 Chandra Family Department of Electrical and Computer Engineering, The University of Texas, Austin, TX 78712 USA

**Keywords:** Quantum optics, Imaging and sensing, Integrated optics

## Abstract

Optomechanical transduction harnesses the interaction between optical fields and mechanical motion to achieve sensitive measurement of weak mechanical quantities with inherently low noise. Lithium niobate combines low optical loss, strong piezoelectricity, a high intrinsic *f* × *Q*_*m*_ factor, and low thermal conductivity, making it promising for exploring optomechanical platforms targeting thermal sensing applications. Here, we demonstrate an integrated optomechanical platform on thin-film lithium niobate with precisely engineered optical, mechanical, and thermal fields within a compact 40 μm-diameter footprint. The platform integrates suspended microring resonators with ultrathin central membranes, reducing effective mass and thermal capacitance while maintaining a high intrinsic optical quality factor *Q*_*o*_ of 10^6^ and a mechanical quality factor *Q*_*m*_ of 1117, which increases to 5.1 × 10^4^ upon optomechanical oscillation. The two-step etching suppresses thermal dissipation into the silicon substrate and enhances thermal sensitivity, achieving a temperature coefficient of frequency of −124 ppm K^−1^, a measurable loaded thermal response time of 7.2 μs, and a noise-equivalent power of 6.2 nW Hz^−1/2^ at 10 kHz and room temperature. This compact and scalable platform opens new opportunities for high-sensitivity thermal sensing and power detection and supports heterogeneous integration with infrared absorbers for uncooled infrared detection, thereby paving the way toward large-format infrared sensing arrays with low-noise, all-optical readout.

**Figure:**
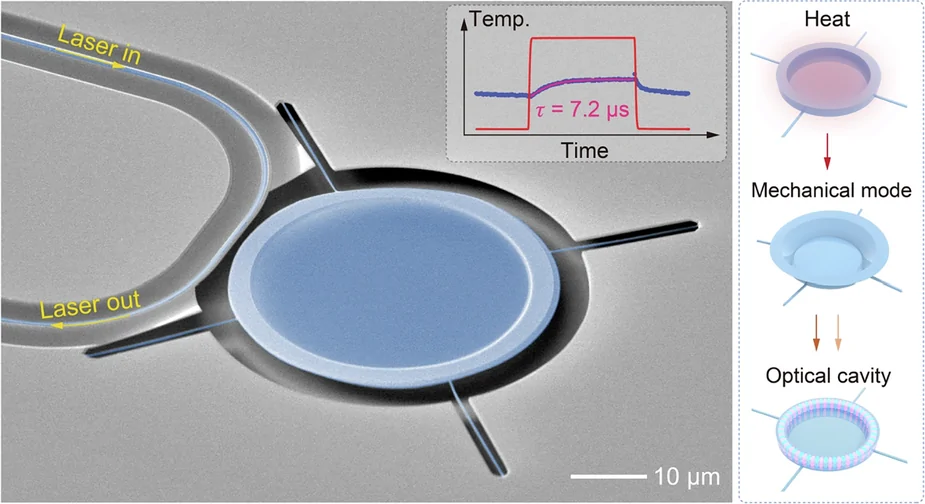
Through co-design of the thermal, mechanical, and optical domains, we demonstrate low-noise sensing with a microsecond-scale response time at room temperature in air

Through co-design of the thermal, mechanical, and optical domains, we demonstrate low-noise sensing with a microsecond-scale response time at room temperature in air

## Introduction

Advances in nanofabrication have enabled the integration of diverse functionalities onto a single chip, yielding smaller, faster, more cost-effective, and energy-efficient platforms. With a tightly confined field and reduced mode volume, integrated platforms facilitate efficient transduction across different physical domains. Cavity optomechanics, which leverages resonant optical confinement to enhance interactions with mechanical motion, has emerged as a powerful paradigm for the sensitive detection and control of weak displacements^1^, underpinning applications in high-precision sensing^2,3^, multiphysical transduction^4,5^, and quantum technologies^6^. The performance of these systems is jointly governed by three key figures of merit: the optical quality factor *Q*_*o*_, which determines the cavity photon lifetime and readout sensitivity; the mechanical *f*⋅*Q*_*m*_ product, which sets the thermomechanical noise floor and mechanical coherence time; and the optomechanical coupling rate *g*_OM_, which quantifies the transduction efficiency between optical and mechanical degrees of freedom. Over the past two decades, on-chip cavity optomechanics has advanced rapidly across multiple material platforms. Silica microtoroid resonators established the foundational principles of radiation-pressure-driven optomechanics with *Q*_*o*_ > 10^8^^7,8^; Silicon-based platforms subsequently leveraged mature CMOS-compatible fabrication to demonstrate optomechanical crystals with *g*_OM_/2*π* > 900 kHz and quantum ground-state cooling^9^, alongside microdisk/ring resonators with *Q*_*o*_ ~ 10^5^−10^6^ and *g*_OM_/2*π* ~ 10−100 kHz for practical sensing applications^10,11^; Silicon nitride (SiN) extended these advances through its low optical absorption (*Q*_*o*_ > 10^7^^12^) and large tensile stress, enabling dissipation dilution in soft-clamped membranes (*Q*_*o*_ > 10^8^^13^) and strain-engineered nanobeams (*f*⋅*Q*_*m*_ > 10^15^^14^). More recently, lithium niobate (LN) has emerged as a compelling platform, uniquely combining low optical loss^15,16^, strong piezoelectricity (*d*_33_ ~ 6 pC N^−1^), large second-order optical nonlinearity^17^, and an intrinsically high *f*⋅*Q*_*m*_ product of 7.1 × 10^13^^18^. These properties have driven breakthroughs in electro-optic modulators, frequency combs, and photonic quantum networks^19–25^. On the optomechanical front, piezo-optomechanical racetrack resonators on thin-film LN (TFLN) have demonstrated *Q*_*o*_ > 10^6^ and *f*⋅*Q*_*m*_ > 10^13^^26^, while suspended LN microdisk/ring resonators, which offer a favorable balance between compact footprint, strong optomechanical coupling, and fabrication simplicity, have achieved *Q*_*o*_ of 2.3 × 10^5^ and *g*_OM_/2*π* of 11.2 kHz with central pedestal support^27^. Combined with LN’s large thermal expansion coefficient (16.7 ppm K^−1^) and low thermal conductivity (4.6 W m^−1^ K^−1^), these properties make TFLN a uniquely attractive foundation for sensitive, room-temperature optomechanical thermal sensing.

Uncooled bolometers and thermal sensors represent one of the most compelling applications of cavity optomechanics, wherein absorbed radiation raises the resonator temperature and shifts its mechanical resonance frequency via the temperature coefficient of frequency (TCF). Maximizing sensing performance requires simultaneous optimization across thermal, mechanical, and optical domains: a large thermal expansion coefficient to enhance TCF; a low thermal conductance *G*_th_ for large responsivity (*R* ∝ *TCF*/*G*_th_); a small thermal time constant *τ* = *C*_th_/*G*_th_ for fast detection; and a low mechanical and optical loss to minimize thermomechanical and readout noise. Prior works have addressed these requirements through distinct structural and material strategies, each with inherent trade-offs. Thin membrane geometries have been widely explored to minimize thermal mass *C*_th_ to enhance responsivity, with demonstrations spanning AlN membrane (TCF = −63 ppm K^−1^, *τ* = 0.17 ms)^28^, Si membrane (TCF = −70 ppm K^−1^, *τ* = 0.5 ms)^29^, and low-stress SiN trampoline (TCF = −1065 ppm K^−1^, *τ* = 12 ms)^30^. However, these approaches share a fundamental limitation: since *G*_th_ and *C*_th_ both scale proportionally with membrane thickness, *τ* remains unchanged upon membrane thinning, constraining thermal bandwidth to the millisecond regime. SAW-based resonators on piezoelectric substrates achieve high acoustic frequencies and electrical readout, with a TCF of −75 ppm K^−1^ on LN SAW^31^ and −31.5 ppm K^−1^ on AlN SAW^32^, but are fundamentally limited by the *h*/*λ* ratio and poor substrate thermal isolation. At the extreme of dimensional scaling, low-dimensional resonators, including 2D materials such as MoS_2_ and 1D nanowire structures, exhibit giant TCF values of 10^3^−10^4^ ppm K^−1^ arising from their extreme surface-to-volume ratio and boundary-condition-dominated elastic response^33^. However, their atomically thin geometry precludes on-chip optical readout, necessitating free-space optical or electrical detection schemes that severely compromise integration and scalability.

Here, we demonstrate room-temperature, low-noise optomechanical sensors on TFLN that resolve the fundamental trade-offs limiting prior platforms through three key innovations. First, LN provides a uniquely favorable material foundation for thermal sensing: its large thermal expansion coefficient of 16.7 ppm K^−1^ (2.6 ppm K^−1^ for Si and 3 ppm K^−1^ for SiN) directly yields a TCF of −124 ppm K^−1^ that exceeds what is achievable on conventional platforms, and its small thermal conductivity of 4.6 W m^−1^ K^−1^ (148 W m^−1^ K^−1^ for Si and 2.5 W m^−1^ K^−1^ for SiN) establishes a large responsivity floor. Second, the bowl-shaped suspended resonator geometry decouples the optimization of thermal, optical, and mechanical performance within a single compact 40 μm diameter pixel. Selectively trimming the inner membrane from 300 nm to 80 nm largely reduces *C*_th_ without proportionally affecting *G*_th_, breaking the *G*_th_-*C*_th_ coupling and pushing intrinsic *τ* to 5 μs (loaded *τ* of 7.2 μs in measurement), over two orders of magnitude faster than state-of-the-art membrane sensors. Third, the thick outer rim supported by ultrathin, narrow anchors simultaneously minimizes optical and mechanical loss while thermally isolating the resonator from the silicon substrate, preserving responsivity. This geometry achieves an intrinsic optical quality factor *Q*_*o*_ of 1 × 10^6^, a mechanical quality factor *Q*_*m*_ of 1117 at room temperature and ambient pressure, and a strong optomechanical coupling rate *g*_OM_/2*π* of 4.2 kHz, enabling fully integrated all-optical readout and eliminating the free-space and electrical detection schemes that limit prior platforms. Together, our design minimizes fundamental noise contributions, including thermal fluctuation noise, thermomechanical noise, and optical readout noise, yielding a noise equivalent power (NEP) of 6.2 nW Hz^−1/2^ at room temperature and ambient pressure. These results establish a scalable, low-noise platform for room-temperature optomechanics as the backbone for precision thermal sensing with frequency-shift modality and all-optical readout. Heterogeneous integration with thin-film infrared absorbers^34–36^ further extends the platform toward large-scale, spectrally multiplexed, uncooled infrared focal plane arrays, opening new regimes of sensitivity and imaging capability inaccessible to prior optomechanical and membrane-based thermal sensing approaches.

## Results

**Fig. 1:**
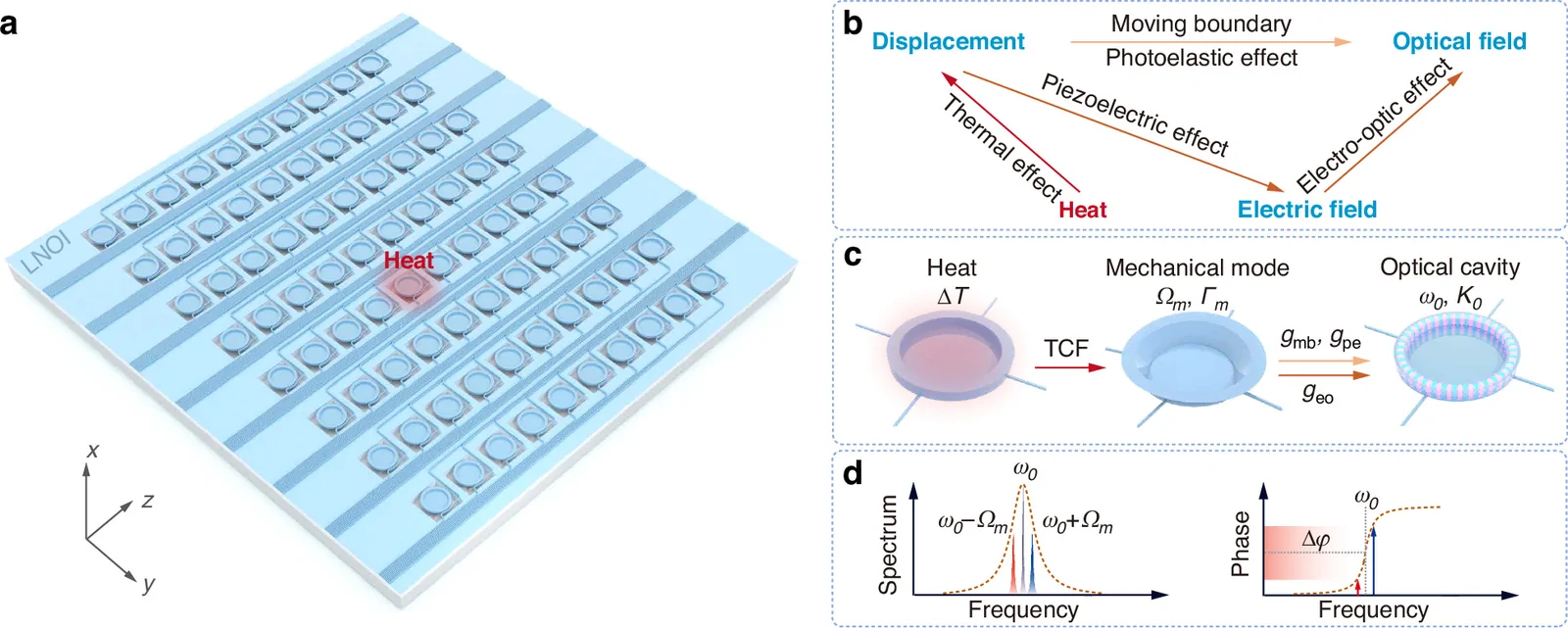
**Room-temperature optomechanical platform on TFLN**. **a** Illustration of the bowl-shaped optomechanical resonator. **b** Thermal sensing based on optomechanical transduction on LN. **c** Transduction between thermal, mechanical, and optical fields with labeled parameters: mechanical frequency and damping rate (Ω_*m*_, Γ_*m*_), optical frequency and damping rate (*ω*_*o*_, *κ*_*o*_), coupling rate induced by moving boundary, photoelastic, and electro-optic effects (*g*_*mb*_, *g*_*pe*_, *g*_*eo*_). **d** Transduction mechanism for the laser resonantly probing the cavity, where the mechanical motion causes the reflected field to be phase modulated

Figure 1a illustrates our low-noise optomechanical platform fabricated on TFLN, where hundreds of pixels are integrated on a 1cm × 1cm chip. On optomechanical platforms like silicon and silicon nitride, mechanical displacement can be directly coupled to the optical field through the moving-boundary (MB) and photoelastic (PE) effects. Due to the strong electro-optic (EO) and piezoelectric effects of LN, an additional optomechanical coupling path arises, in which mechanical displacement introduces an internal electric field through the piezoelectric effect that can further modulate the optical field through the EO effect (Fig. 1b). Heat accompanied by a temperature change *ΔT* is transduced to a mechanical frequency* Ω*_*m*_ shift via the thermomechanical effect. This frequency shift is subsequently read out in the optical domain with a total single-photon optomechanical coupling rate *g*_0_ = *g*_0,*MB*_ + *g*_0,*PE*_ + *g*_0,*EO*_, defined as the frequency shift in the optical mode due to the zero-point motion of the mechanical oscillator^26^ (Fig. 1c). With a zero-detuning optical pump coupled into the resonator, the thermomechanical vibration strongly modulates the optical phase, which can be precisely measured using balanced homodyne detection at the output (Fig. 1d).

**Fig. 2:**
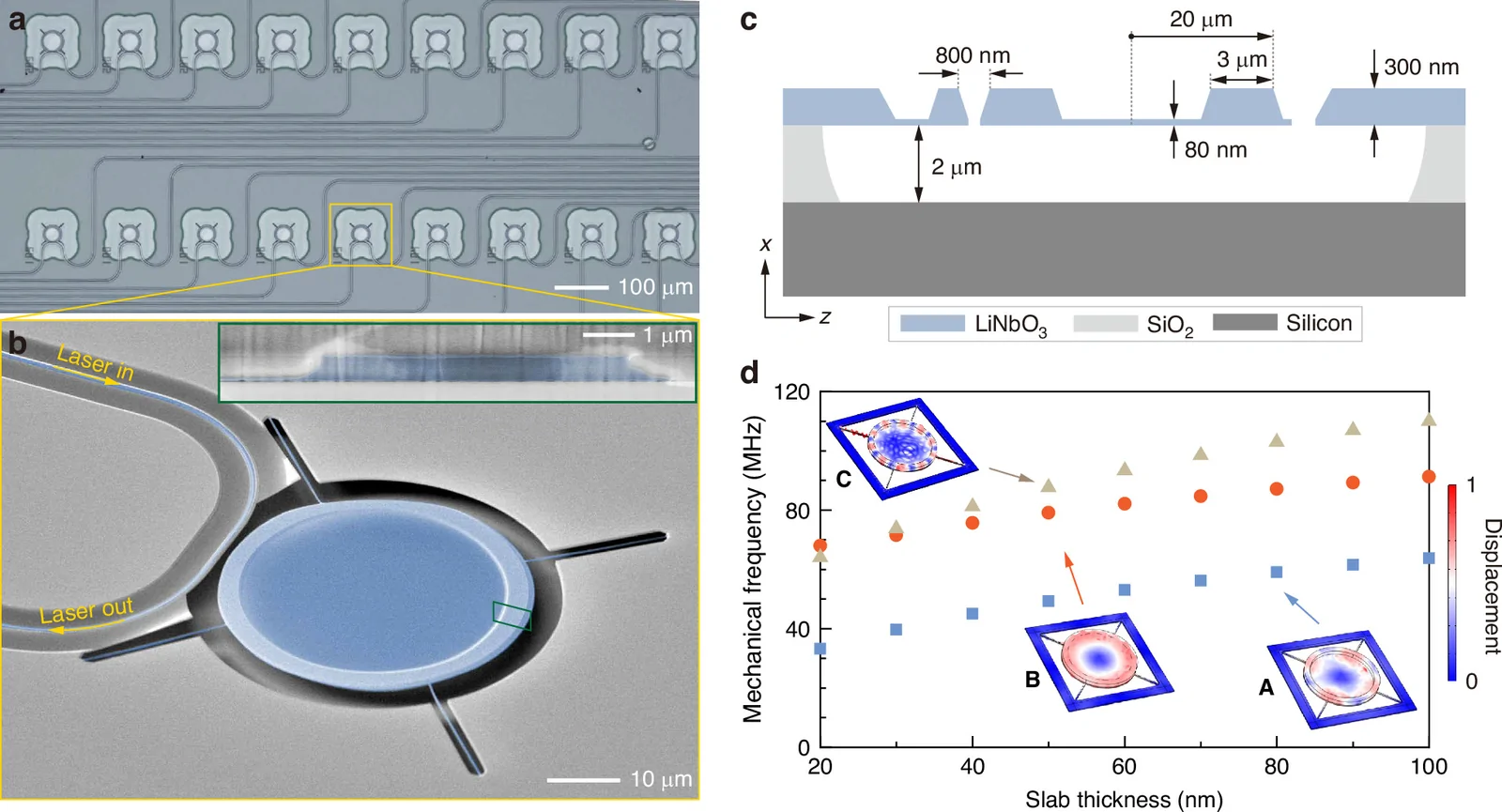
**Design and numerical simulation of bowl-shaped optomechanical resonators**. **a** A microscope image of arrays of optomechanical pixels, demonstrating large scalability. **b** SEM images of a fabricated resonator; the inset shows the cross-section. **c** Side views of the device, with dimension labels. **d** Simulated mechanical frequency of three modes at different slab thicknesses. The insets represent the mechanical displacement profiles of corresponding modes

To realize a low-noise optomechanical platform for future sensing applications, high-quality optical and mechanical resonances are essential to suppress thermal and readout noise and maintain coherence, along with a strong optomechanical coupling rate for efficient transduction between optical and mechanical degrees of freedom. We designed radial-mode bowl-shaped optomechanical resonators on 300-nm X-cut LN on insulator (Fig. 2a), which support tightly confined optical modes circulating around their outer circumference, and strong mechanical vibrations enabled by a reduced inner membrane thickness (Fig. 2b). Figure 2c shows the cross-section of our device with labeled dimensions. The resonator has outer and inner radii of 20 and 17 μm to balance the optical bending loss and the footprint of the device. Four anchors, each 400 nm wide and 20 μm long, are fabricated on the slab layer and attached to the outer rim to provide mechanical support as well as minimize the perturbation of both optical and mechanical confinement. By balancing the device performance and fabrication challenges, we chose the anchor (and slab) thickness of 80 nm for fabrication, which included two-step e-beam lithography and etching, annealing, and suspension (see Materials and methods for details). The small volume of the anchors is optimized to reduce the thermal conductance, thereby enhancing thermal sensitivity and making them suitable for on-chip bolometer applications. A bus waveguide is placed in close proximity to the resonator to couple light into and out of the resonator for detecting the thermomechanical vibration via optomechanical transduction. Next, we simulate the frequencies of supported mechanical modes at different thicknesses of the inner membrane (*t*_slab_). Figure 2d only plots the fundamental frequency of three mechanical modes (A, B, C) near 80 MHz with their corresponding displacement profiles (embedded). Although high *f* × *Q*_*m*_ products can be achieved in higher-order mechanical modes^37,38^, their proximity to numerous neighboring modes leads to elevated displacement background noise. For this reason, optimal low-noise operation is achieved by preferentially coupling to the fundamental mechanical mode of the resonator.

**Fig. 3:**
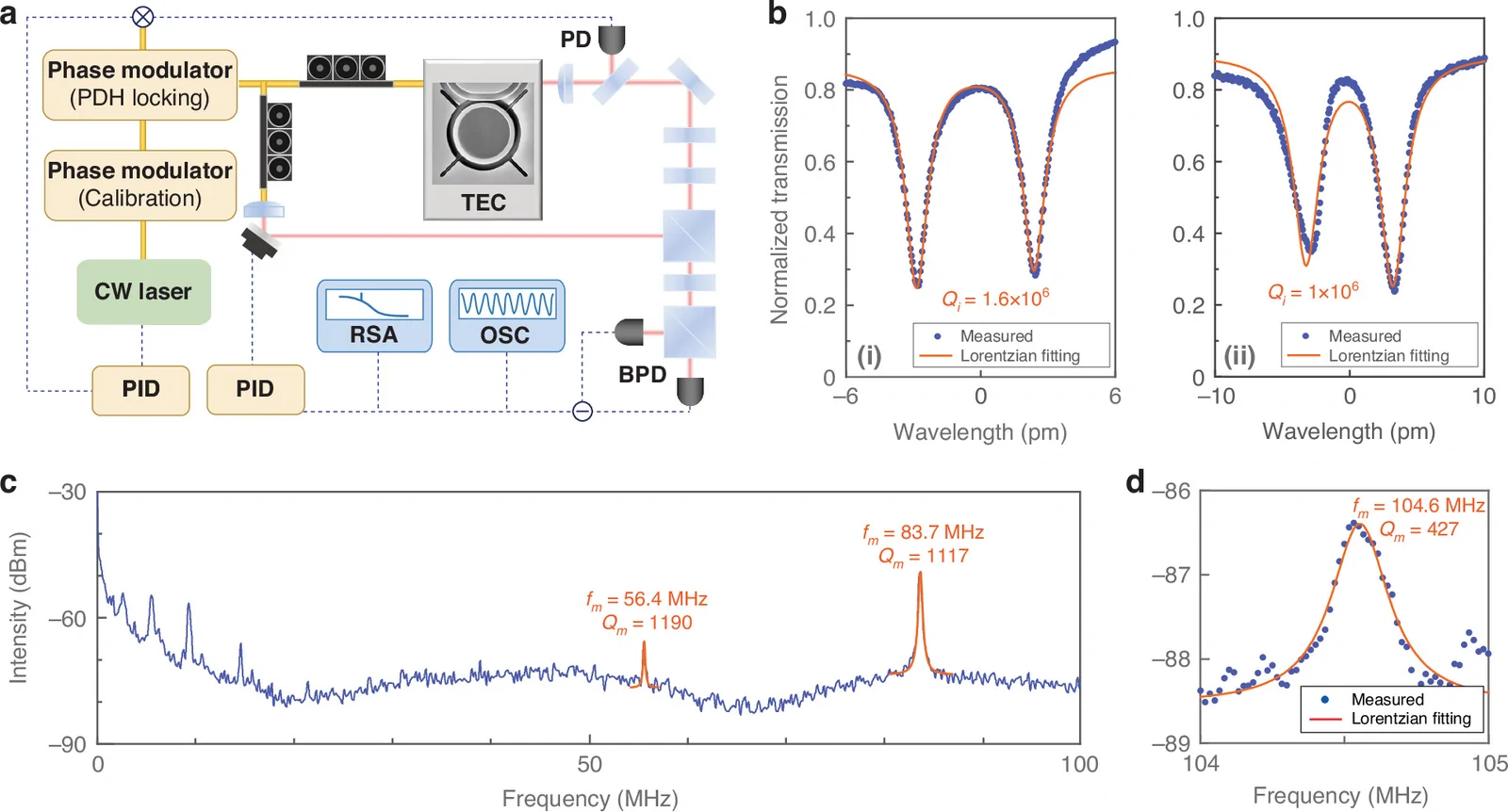
**Experimental setup and optomechanical characterization**. **a** Experimental setup, CW continuous wave, PDH Pound-Drever–Hall, PID Proportional-Integral-Derivative controller, TEC thermoelectric cooler, PD photodetector, BPD balanced photodetector, RSA signal analyzer, OSC oscilloscope. **b** Measured optical resonance with Lorentzian-fitted optical *Q*_*i*_ factors after first etching (i) and suspension (ii). **c** Measured PSD through phase detection up to 100 MHz. **d** Measured PSD through intensity detection near 104 MHz

Figure 3a shows the experimental setup to measure the thermomechanical vibration using balanced homodyne detection with the assistance of the Pound–Drever–Hall locking (see Materials and methods for details). This scheme is used to suppress the laser-cavity detuning noise and the laser intensity noise to achieve a stable and precise optical readout. All measurements were conducted at room temperature and atmospheric pressure, unless otherwise noted. To reduce the etching-induced optical loss, the anchors are positioned away from the outer rim of the resonator (see Supplementary Sec. 1). This strategy allows us to maintain low optical loss even after two-step etching, where the fabricated device shows an intrinsic quality factor *Q*_*o*_ of 1.6 × 10^6^ and 1 × 10^6^ after the first etch (i) and the final suspension (ii), as shown in Fig. 3b. The decreased resonance extinction ratio after suspension is mainly attributed to the vertical movement of the suspended resonator, which becomes more pronounced as the anchors become thinner, while most of the bus waveguide is still supported on the substrate. Note that the resonance doublets are consistently observed for the fundamental TE_0_ modes, which originate from backscattering-induced mode splitting within the same TE_0_ mode family. We then measure the displacement noise power spectral density (PSD) of mechanical modes using phase detection for frequencies below 100 MHz and intensity detection for frequencies above 100 MHz (Fig. 3c, d). With an on-chip power of 56 μW and a local-oscillator power of 388 *μ*W, a maximum signal-to-noise ratio of 24 dB is achieved at 83.7 MHz with *Q*_*m*_ of 1117, corresponding to the fundamental radial mode B shown in Fig. 2d.

**Fig. 4:**
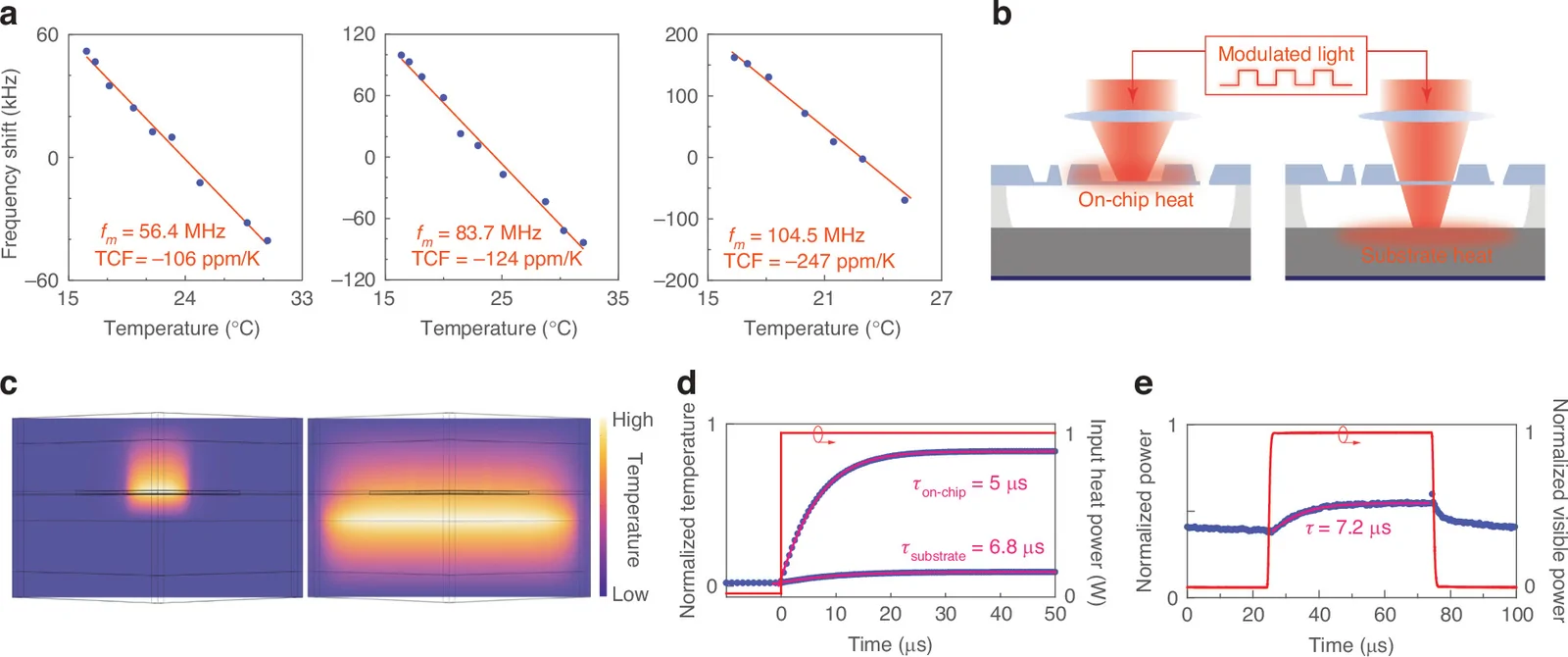
**Thermal sensitivity and dynamical response characterization**. **a** Measured TCFs for three mechanical modes at 56.4 MHz, 83.7 MHz, and 104.5 MHz, extracted from linear fits (orange lines) to the frequency shift versus temperature data (blue dots). **b** Schematic of the two configurations used for dynamical thermal response characterization: on-chip heating (left) and substrate heating (right). **c** Finite-element simulated steady-state temperature distributions for on-chip heating (left) and substrate heating (right). **d** Simulated time-domain temperature response (blue dots) of the resonator under a step heat input (red curve) with logistic fits (pink curve). **e** Measured normalized output power (blue dots) in response to a square-wave visible light input (red curve), with a logistic fit (pink curve)

We then characterize the thermal sensitivity of the device by tuning the equilibrium temperature from 289 to 306 K using thermoelectric coolers (TECs) (see Supplementary Sec. 2). Our resonator shows high TCF values ranging from −106 to −263 ppm K^−1^ for different mechanical modes, as shown in Fig. 4a (also see Supplementary Sec. 3). The fundamental mode located at 83.7 MHz has a TCF of −124 ppm K^−1^, exceeding those of other integrated platforms (∣*TCF*∣ < 50 ppm K^−1^^39^). In suspended optomechanical resonators, the intrinsic *G*_th_ is governed by device geometry. Our two-step etching strategy substantially reduces *C*_th_ while increasing *G*_th_, thereby enhancing the thermal response speed. However, the measured *G*_th_ is also influenced by the position and size of the heat source: a heat source located away from the resonator introduces an additional spreading thermal resistance that retards the overall response. To elucidate the dynamical behavior of the sensor, we consider two representative configurations in which the heat source is positioned either across the entire resonator or on the top surface of the silicon substrate (Fig. 4b). In finite-element simulations, the surrounding air and the bottom surface of the silicon substrate are set to 293 K, a step heat input of 1 W is applied, and the temperature change at the ring rim is mointored. Figure 4c shows the steady-state temperature distributions, where the resonator reaches a higher equilibrium temperature in both configurations. Figure 4d presents the simulated time-domain responses together with logistic fits, yielding intrinsic *τ* = 5 μs for heating on the resonator and loaded *τ* = 6.8 μs for heating on the silicon substrate. The modest increase in *τ* for the substrate configuration is attributed to the additional thermal pathway from the substrate through the surrounding medium to the resonator. Notably, substrate-heating also produces a substantially smaller temperature rise, indicating a reduced thermal responsivity. We also examine how membrane thickness affects the response: thinning the inner membrane from 300 to 80 nm reduces *τ* from 8.4 *μ*s to 5 *μ*s, confirming the advantage of the two-step etching design (Supplementary Section 4). In the absence of an efficient on-chip light absorber, we exploit the visible-wavelength absorption of the silicon substrate to characterize the sensor’s dynamical response experimentally. A square-wave intensity modulation is imposed on the incident visible light using an amplitude modulator, and the modulated beam is focused onto the device. Fitting the optical output waveform yields a loaded *τ* = 7.2 μs (Fig. 4e), in good agreement with our simulation results. Integration of an on-chip optical absorber will enable direct excitation with higher responsivity, providing a more accurate characterization of both response speed and thermal sensitivity.

**Fig. 5:**
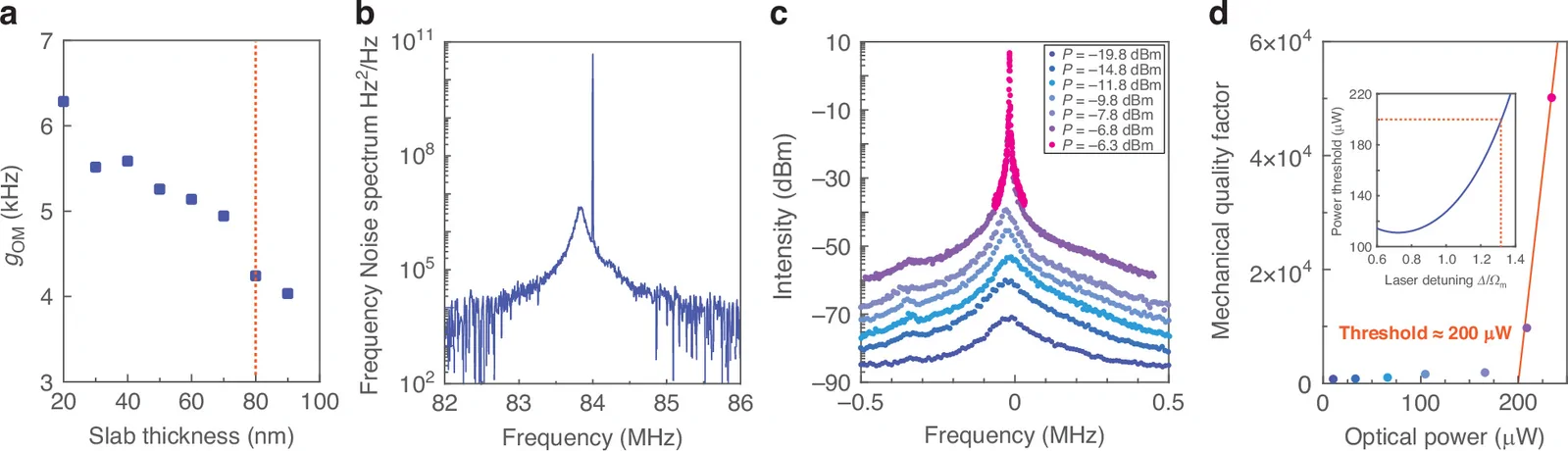
**Optomechanical coupling characterization**. **a** Simulated optomechanical coupling rate at different slab thicknesses, showing a *g*_OM_ of 4.2 kHz at a slab thickness of 80 nm. **b** Applied calibration peak at 84 MHz, near the target frequency, used to extract the optomechanical coupling rate. **c** Measured mechanical amplification as a function of increasing optical power, showing a drastic reduction in linewidth accompanied by a pronounced increase in oscillation intensity when exceeding the power threshold. **d** Measured *Q*_*m*_ at different optical powers with the inset showing the laser-detuning-dependent lasing threshold

We further characterize the single-photon optomechanical coupling rate *g*_0_ of the fundamental radial mode by adding another phase modulation signal to the input light and generating the calibration sideband near the target frequency, as shown in Fig. 5b. By comparing the calibration sideband power with the mechanical sideband power, we obtain the experimental *g*_OM_ of 4.2 kHz, which is consistent with the simulation result shown in Fig. 5a at an 80-nm slab thickness. The performance of the optomechanical resonator can also be quantified by measuring the mechanical linewidth as a function of pumping power. Figure 5c plots measured RF spectra of cavity transmission at different pump powers. When the pump power is below the oscillation threshold, the mechanical spectrum closely resembles that of thermal mechanical vibration, as shown in Fig. 5b. As the pump power increases, the mechanical mode is driven into an oscillation state, and the peak value of the mechanical spectral intensity is enhanced by 80 dB. Simultaneously, the mechanical linewidth is drastically narrowed to 1.6 kHz, corresponding to an effective *Q*_*m*_ of 5 × 10^4^ (Fig. 5d). This behavior clearly demonstrates mechanical lasing, and by fitting the above-threshold data with a linear curve, we extract a threshold power of 200 μW. The power threshold for optomechanical lasing is given by^40^

1 $${P}_{{{{\rm{th}}}}}=\dfrac{{m}_{{{{\rm{eff}}}}}{\omega }_{o}{{{\Gamma }}}_{i}{{{\Gamma }}}_{m}}{2{g}_{{{{\rm{OM}}}}}^{2}{{\Gamma }}\Delta }\left[{(\Delta -{{{\Omega }}}_{m})}^{2}+{({{\Gamma }}/2)}^{2}\right]\left[{(\Delta +{{{\Omega }}}_{m})}^{2}+{({{\Gamma }}/2)}^{2}\right]\dfrac{4{\Delta }^{2}+{{{\Gamma }}}^{2}}{4{{{\Gamma }}}_{i}{{{\Gamma }}}_{e}}$$

where *P*_th_ is the on-chip threshold optical power, *m*_eff_ is the effective motional mass, Γ_*i*_, Γ_*e*_, and Γ are the intrinsic, external, and total photon decay rates, respectively. Γ_*m*_ is the energy decay rate of the mechanical mode and *Δ* is the laser-cavity detuning. Using the measured resonator parameters, we can derive the theoretical power threshold of the device at different detunings, as shown in the inset of Fig. 5d. This indicates that our experimental detuning is around 1.3 Ω_*m*_, which is not the optimal value due to the non-optimized laser-cavity detuning used in the experiment. Further optimization of the laser-cavity detuning would enable an optimal power threshold of 110 μW.

Noise in an optomechanical system can limit its performance in sensing, timing, and transduction applications. We now analyze the noise sources in our optomechanical system and quantify them using NEP. Thermal fluctuation noise NEP_*T*_, thermomechanical displacement noise NEP_thermo_, and optical readout noise NEP_opt_ are the three fundamental noise sources on our platform. Note that the large SNR value measured in our experiment indicates that the optical readout noise contribution to frequency uncertainty is negligible compared to the thermomechanical noise floor. The other two noise sources can be written as^29^

2 $${{{{\rm{NEP}}}}}_{T} =\dfrac{1}{{{{\rm{TCF}}}}}\,\dfrac{df}{dT}\sqrt{\dfrac{4{k}_{B}{T}^{2}{G}_{{{{\rm{th}}}}}}{{\omega }^{2}{\tau }_{c}^{2}+1}}\quad \,{\mbox{and}}\,\quad {{{{\rm{NEP}}}}}_{{{{\rm{thermo}}}}}\\ =\dfrac{{G}_{{{{\rm{th}}}}}}{{{{\rm{TCF}}}}}\,\dfrac{df}{dx}\sqrt{\dfrac{{k}_{B}T}{{m}_{{{{\rm{eff}}}}}\,{\omega }_{m}^{2}\,{Q}_{m}}}$$

where *T*, *G*_th_, *τ*_*c*_, *ω*_*m*_, and *m*_eff_ are the absolute temperature, thermal conductance, time constant, mechanical resonant frequency, and effective mass, respectively. Note here *τ*_*c*_ = *C*/*G*_th_, where *C* is the thermal capacitance. With well-engineered thermal mass and heat dissipation, the device achieves a simulated *G*_th_ of 108 μW K^−1^ and a small *C* of 540 pJ K^−1^, corresponding to a *τ*_*c*_ of 5 *μ*s and a thermal bandwidth of 31.8 kHz. Further decreasing *G*_th_ can reduce both NEP_*T*_ and NEP_thermo_, while increasing TCF and *Q*_*m*_ can reduce NEP_thermo_ (see also Supplementary Sec. 5).

**Fig. 6:**
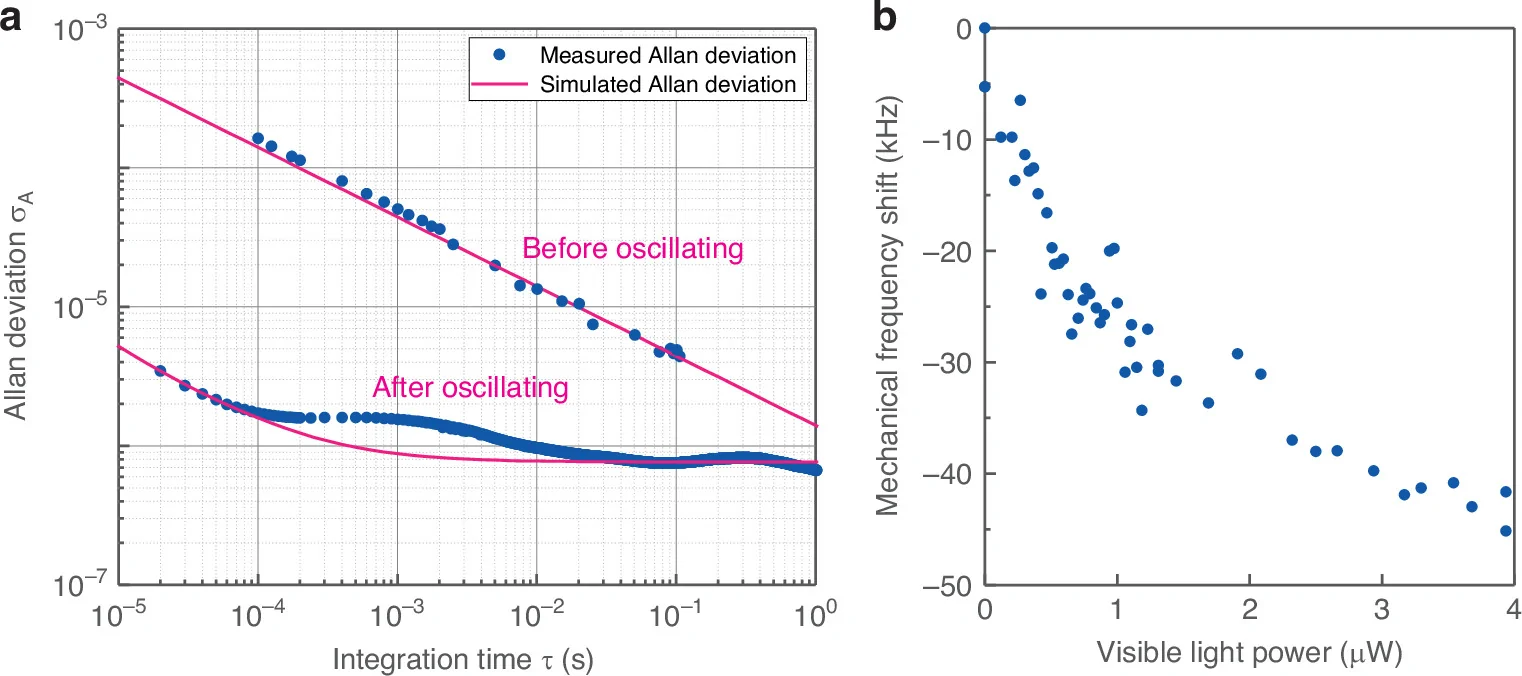
**Allan deviation measurement and power detection application**. **a** Measured *σ*_*A*_ before and after oscillation. Blue dots denote the experimental data, while pink lines show the corresponding fits. Before oscillation, frequency instability is dominated by thermomechanical noise, yielding *σ*_*A*_ ∝ *τ*^−1/2^. After oscillation, the overall noise reduces, but the scaling deviates at longer integration times due to the emergence of flicker noise and random-walk frequency noise. **b** Measured mechanical frequency response to the incident visible power. The measurement is taken with 20 *μ*W of telecom power on-chip to avoid additional thermal effects

We characterize the frequency stability of the device using Allan deviation (*σ*_*A*_), which is subsequently converted to NEP via the device responsivity. Figure 6a compares the measured *σ*_*A*_ versus integration time *τ* before and after oscillation. Before oscillation, due to the relatively low signal intensity ( ~ − 48 dBm), we use an oscilloscope to capture the output signal and extract the Allan deviation (see Materials and methods). In this case, the upper limit of measurable *τ* is constrained by the memory depth of the oscilloscope. Within the measured range, *σ*_*A*_ scales as *τ*^−1/2^, indicating that thermomechanical noise dominates the frequency instability. A *σ*_*A*_ of 1.6 × 10^−4^ is obtained at an integration time of 10^−4^ s, corresponding to a NEP of 610 nW Hz^−1/2^. After oscillation, the signal intensity increases significantly, allowing direct detection with a commercial frequency counter. At a short integration time, thermomechanical noise remains the primary contributor, yielding a *σ*_*A*_ of 1.6 × 10^−6^ at *τ* = 10^−4^ s, corresponding to a NEP of 6.2 nW Hz^−1/2^. At longer timescales, flicker noise and random-walk noise become dominant (see Supplementary Fig. S5), which might originate from laser instability at high optical power and can be suppressed by adding balanced homodyne detection or PDH locking. Operating the device under vacuum can further reduce the thermal conductance by three orders of magnitude, substantially lowering the noise floor. In addition, optimizing the anchor structure or introducing a feedback loop^12,41–43^ may further enhance sensitivity and suppress noise (also see Supplementary Section 5).

The optimized thermal performance also enables sensitive optical-power detection. To demonstrate this capability for practical applications, we illuminate the suspended-resonator region with 780-nm light using the same experimental configuration employed for the thermal-response measurements. Figure 6b shows the measured mechanical-frequency shift as a function of incident optical power. Below 1 μW, the response is approximately linear, with a power-to-frequency responsivity $| \frac{{{{\rm{d}}}}{f}_{{{{\rm{m}}}}}}{{{{\rm{d}}}}{P}_{{{\mathrm{inc}}}}}|$ of 30 kHz μW^-1^ ; at higher powers, the frequency shift gradually saturates, defining the linear operating range of the present device. The current implementation is wavelength-selective and produces no measurable response at telecom or mid-infrared wavelengths. Broader spectral coverage could be achieved by integrating a patterned absorber directly onto the suspended membrane^44^.

Together, we successfully established the TFLN optomechanical resonator as a compact platform for low-noise, rapid detection of temperature and optical power through an integrated, low-loss optical readout. Compatibility with remote fiber-based interrogation may further support stable, long-distance sensing in hazardous or inaccessible environments, such as underground infrastructure and oil-and-gas facilities.

## Discussion

We have demonstrated a compact, room-temperature optomechanical sensing platform on TFLN that co-localizes a high-*Q* optical cavity and a high-frequency mechanical resonator within a 40 μm-diameter footprint. The bowl-shaped resonator maintains an optical quality factor of *Q*_o_ = 1 × 10^6^ while supporting an approximately 84 MHz mechanical mode with *Q*_m_ = 1117. The resulting *f* ⋅ *Q* ~ 10^11^ Hz is comparable with state-of-the-art anchor-supported architectures operating in air^45–48^. Self-oscillation increases the effective mechanical quality factor to 5.1 × 10^4^, enabling low-noise frequency tracking through the integrated optical readout. This combination of compactness and optical and mechanical performance underpins the temperature- and optical-power-sensing capabilities of the platform.

Table 1 benchmarks room-temperature thermal sensors and bolometers based on thermomechanical and thermo-optic transduction. Our device supports both mechanisms, with a TOC of 37.3 pm K^−1^ and a TCF of −124 ppm K^−1^. Across the surveyed thermo-optic sensors, the wavelength responsivity spans approximately 4.1 to 770 pm K^−1^, reflecting differences in material response, optical confinement, and spectral engineering. Fano-resonant spectral engineering can enhance wavelength responsivity^49^, whereas resonance locking and self-referenced schemes can improve stability and temperature resolution^50,51^. These techniques, however, require either spectral acquisition, multiple-mode tracking, or feedback electronics, so their system bandwidth or sensitivity is not determined by the intrinsic thermal response alone. Without Fano-type spectral enhancement, our LN resonator exhibits a TOC of 30 pm K^−1^ and a 5 μs thermal time constant. This is the shortest response time within the surveyed thermo-optic sensors operating in air. Thermomechanical transduction instead maps the temperature-induced frequency shift onto an electronically-accessible MHz carrier, enabling continuous tracking without repeated optical-spectrum acquisition. Our TCF exceeds those reported for integrated platforms, including Si, SiN, SiC, and AlN^39^. Although stress-engineered membranes^29,30,33^ can achieve larger TCF values, their limited optical confinement is a challenge for integrated optical readout.

**Table 1:** Comparison of room-temperature thermal sensors and bolometers

Structure	TCF	TOC	*G*_th_	*τ*	NEP	*Q*_o_	Linewidth shift	Footprint	Pressure	Readout	Integration
	(ppm K^−1^)	(pm K^−1^)	(μW K^−1^)	(μs)	(nW Hz^−1/2^)		(lw K^−1^)	(μm^2^)			
*Thermo-mechanical transduction*											
SiN nanowire^57^	−23,000	–	0.34^I^	2	3−5^B^	–	–	4	Vacuum	Optical	✗ Free-Space
SiN trampoline^30^	−1065	–	0.087^I^	12,000	0.0045^P^	–	–	50 × 50	Vacuum	Optical	✗ Fiber Cavity
Graphene trampoline^33^	+350^I^	–	0.0012^I^	26	0.007^P^	–	–	∅6	Vacuum	Optical	✗ Free-Space
Si membrane^29^	−70	–	0.045^I^	500	0.027	–	–	12 × 8.6	Vacuum	Electronic	*✓* Wired
GaAs membrane^58^	−8485	–	90^I^	55	0.09^P^	–	–	100 × 25	Vacuum	Electronic	*✓* Wired
AlN membrane^28^	−63	–	1.1^I^	166	0.55	–	–	20 × 22	Vacuum	Electronic	*✓* Wired
AlN membrane^36^	+23	–	5.3^I^	440	3	–	–	200 × 75	Air	Electronic	*✓* Wired
Bulk LN^59^	−71.2	–	7.7^I^	169	0.04^S^	–	–	48 × 56	Air	Electronic	*✓* Wired
*Thermo-optic transduction*											
Si photonic-crystal ^60^	–	88^I^	9.3^I^	34	0.18^I^	3.9 × 10^4^	2. 2^I^	23 × 16	Air	Optical	✗ Free-Space
Silica WGM resonator ^51^	–	4.1^I^	NR	200,000^I^	0.015	4 × 10^6^	26^I^	∅190	Air	Optical	✗ Free-Space
LN WGM resonator^61^	–	34.4	–	–	–	NR	NR	NR	Air	Optical	✗ Free-Space
LN photonic crystal ^49^	–	770	–	–	–	NR	NR	25 × 24	Air	Optical	✗ Free-Space
Si microring ^62^	–	77	–	–	–	5.2 × 10^4^	2. 6^I^	∅22	Air	Optical	*✓* Waveguide
LN microdisk^50^	–	6.3^I^	–	–	–	2/2.9 × 10^5^	0.8/1. 2^I^	∅50	Air	Optical	✗ Tapred Fiber
LN microring ^63^	–	28.4	267^a^	1500−2000^a^	–	5.6 × 10^5^	10^I^	∅400	Air	Optical	*✓* Waveguide
**This work**	−**124**	**37.3**	**108**	**5**(**7.2**^b^)	**6.2**^S^	**1** × **10**^**6**^	**24**	**∅40**	**Air**	**Optical**	***✓*** **Waveguide**

Our unique combination of optical and mechanical degrees of freedom enables a fast time constant and high sensitivity in an integrated form factor

TOC denotes the resonance-wavelength temperature sensitivity d*λ*/d*T*, rather than the material coefficient d*n*/d*T*. The linewidth shift expresses the same sensitivity in units of the optical linewidth, lw K^−1^ = (d*λ*/d*T*) *Q*_o_/*λ*_0_, evaluated at *λ*_0_ = 1550 nm unless the source specifies otherwise and referenced to the intrinsic linewidth implied by *Q*_o_. *G*_th_ is referenced to absorbed power throughout. S, self-oscillating; P, phase-locked loop; B, undriven Brownian-motion readout; E, external feedback loop; I, inferred from reported quantities.

^a^Obtained under thermo-optic tuning rather than radiation detection; NR not reported; – not applicable.

^b^loaded *τ.*

As a bolometer, our device exhibits a *G*_th_ of 108 μW K^−1^, a 5 μs thermal time constant, and an NEP of 6.2 nW Hz^−1/2^. Some vacuum-operated thermomechanical bolometers achieve lower NEPs, but their response times range from microseconds to milliseconds. These results illustrate the broader sensitivity-speed trade-off: stronger thermal isolation generally improves power sensitivity but slows thermal relaxation unless the thermal capacitance is reduced concurrently. Rather than achieving the lowest reported NEP, our device offers a balanced combination of microsecond response, ambient operation, compact footprint, and integrated optomechanical readout within a single platform.

We believe the compact footprint, material versatility, and scalability of this platform are well suited to rapid temperature monitoring, optical-power stabilization, and remote fiber-interrogated sensing in electrically noisy or inaccessible environments. Integration of wavelength-selective absorbers could fully enable the capabilities of our architecture for mid-infrared spectroscopy, thermal imaging and multi-spectral detection. The RF-domain output also offers a potential route to frequency-division multiplexing of multiple sensing elements, although array-level operation remains to be demonstrated.

Further improvements should target absorption efficiency, thermal isolation and frequency stability simultaneously. Improving *f*⋅*Q* remains an important priority and can be pursued through anchor geometry optimization and vacuum operation to suppress air damping. More broadly, meandered tether geometries^52^ and acoustic bandgap engineering^53^ can reduce the effective thermal mass and push the thermal conductance toward the radiative limit, simultaneously accelerating the thermal response and enhancing sensitivity. Patterned absorbers could increase the fraction of incident power converted into a measurable response and extend the operating wavelength range. Benefiting from the strong piezoelectric response of LN, an external optoelectronic feedback loop can be implemented via electro-mechanical actuation^54^ to suppress phase noise, analogous to techniques previously demonstrated in optoelectronic oscillators^55^. Meanwhile, optical-detuning-sensitive self-oscillation enabled by such feedback provides a pathway to significantly enhance the effective TCF through the thermo-optic effect^56^. Operation under reduced pressure offers an additional route to noise suppression: at an ambient pressure of 1 mbar, *G*_th_ is expected to decrease by three orders of magnitude, leading to a projected NEP as low as 6.2 pW Hz^−1/2^.

## Materials and methods

### Fabrication

The TFLN samples under test are fabricated on an X-cut lithium-niobate-on-insulator wafer from NanoLN with a 300-nm-thick top LN layer and a 2-μm-thick bottom silicon oxide layer on a silicon substrate. Microring resonators and bus waveguides are first patterned in positive resist (PMMA) with electron-beam lithography (EBL) and transferred to the LN using angled argon milling, leaving an 80-nm-thick slab. By optimizing the etching recipe, we significantly reduce the trenching effect, enabling the realization of thinner and more uniform slabs. After stripping the resist, a thorough acid clean is performed to remove re-deposited amorphous LN. A second EBL defines the anchors and air-holes in PMMA, followed by full etching using Ar^+^-based inductively coupled plasma reactive ion etching (ICP-RIE). After repeating the cleaning process, the chips are annealed at 500 ^∘^C for 3 h to restore the LN material quality. Finally, the optomechanical resonators are released by wet etching in 49% hydrofluoric acid and dried in a critical point dryer.

### Measurement

A tunable continuous-wave laser (Santec TSL-570) is coupled into on-chip edge couplers via a lensed fiber and collected by a collimator, with a total coupling loss of 18.8 dB, primarily attributed to facet roughness from hand cleaving. The device under test (DUT) is mounted on a commercial TEC for temperature-dependent measurement. For thermal dynamical measurements, a coaxial free-space setup delivers visible-wavelength illumination onto the device from above, with an electro-optic amplitude modulator imposing a square-wave modulation on the excitation beam. For phase-shift and optomechanical coupling rate measurements, a fiber-based homodyne interferometer is employed. The input laser is split into two paths: one coupled into the resonator to bring out the mechanical signal, while the other serves as a phase-stable local oscillator. Interference between the two paths converts the phase modulation into an intensity signal, which is detected by the balanced photodetectors, enhancing the signal-to-noise ratio. Pound-Drever-Hall locking is used to lock the laser at the resonant wavelength, where the phase response slope is maximal and thus allows the most sensitive phase readout. Mechanical mode spectra are acquired as power spectral densities (PSDs) using a signal analyzer, while an oscilloscope monitors the mechanical frequency stability in the time domain. For Allan deviation measurements below the oscillation threshold, the output optical signal—modulated at the mechanical resonance frequency—is recorded on an oscilloscope over an extended time window. To resolve frequency fluctuations on the order of 10 kHz around the 83.7 MHz carrier, a minimum time window of 1 ms at a sampling rate exceeding 200 MS s^−1^ is required. The recorded waveform is segmented into 1 ms intervals, and a short-time Fourier transform is applied to each segment to extract the instantaneous mechanical frequency as a function of time, from which the Allan deviation is computed at varying integration times. Above the oscillation threshold, a commercial frequency counter directly yields the Allan deviation.

## Supplementary information

Supplementary Information

## Data Availability

The data that support the findings of this study are available from the corresponding author upon reasonable request.
