# Supplementary material for: Uncooled low-noise optomechanical sensors on thin-film lithium niobate
Source: Light Sci Appl. 2026 Oct 20;15:389. doi: 10.1038/s41377-026-02506-z (PMC13683413; doi:10.1038/s41377-026-02506-z)
Supplement: Supplementary file 1 [file 41377_2026_2506_MOESM1_ESM.pdf]

Supplementary Information for "Uncooled low-noise optomechanical sensors on thin film lithium niobate"

Yue Yu,[1,2,3,†] Ran Yin,[1,2,†] Ian Anderson,[4] Yinan Wang,[4] Jack Kramer,[1,4] Chun-Ho Lee,[1,2] Xinyi Ren,[1,2] Zaijun Chen,[1,2] Michelle L. Povinelli,[2] Dan Wasserman,[4] Ruochen Lu,[4] and Mengjie Yu[1,2,3,*]

[1]*Department of Electrical Engineering and Computer Sciences, University of California, Berkeley, Berkeley, CA, 94720, USA.*

[2]*Ming Hsieh Department of Electrical and Computer Engineering, University of Southern California, Los Angeles, CA, 90089, USA.*

[3]*Materials Sciences Division, Lawrence Berkeley National Laboratory, Berkeley, CA, 94720, USA.*

[4]*Chandra Family Department of Electrical and Computer Engineering, The University of Texas at Austin, TX, 78712, USA.*

[†]*These authors contributed equally to this work.*

[*]*Corresponding author: mengjie.yu@berkeley.edu*

1. STRUCTURAL DESIGN OF BOWL-SHAPED OPTOMECHANICAL RESONATORS

To reduce the optical and mechanical loss induced by the four anchors, we used a two-step etching process, as mentioned in the Fabrication Section in the main text. Figure S1 shows more details of the structures after the first (dark blue) and second (light blue) etching steps.

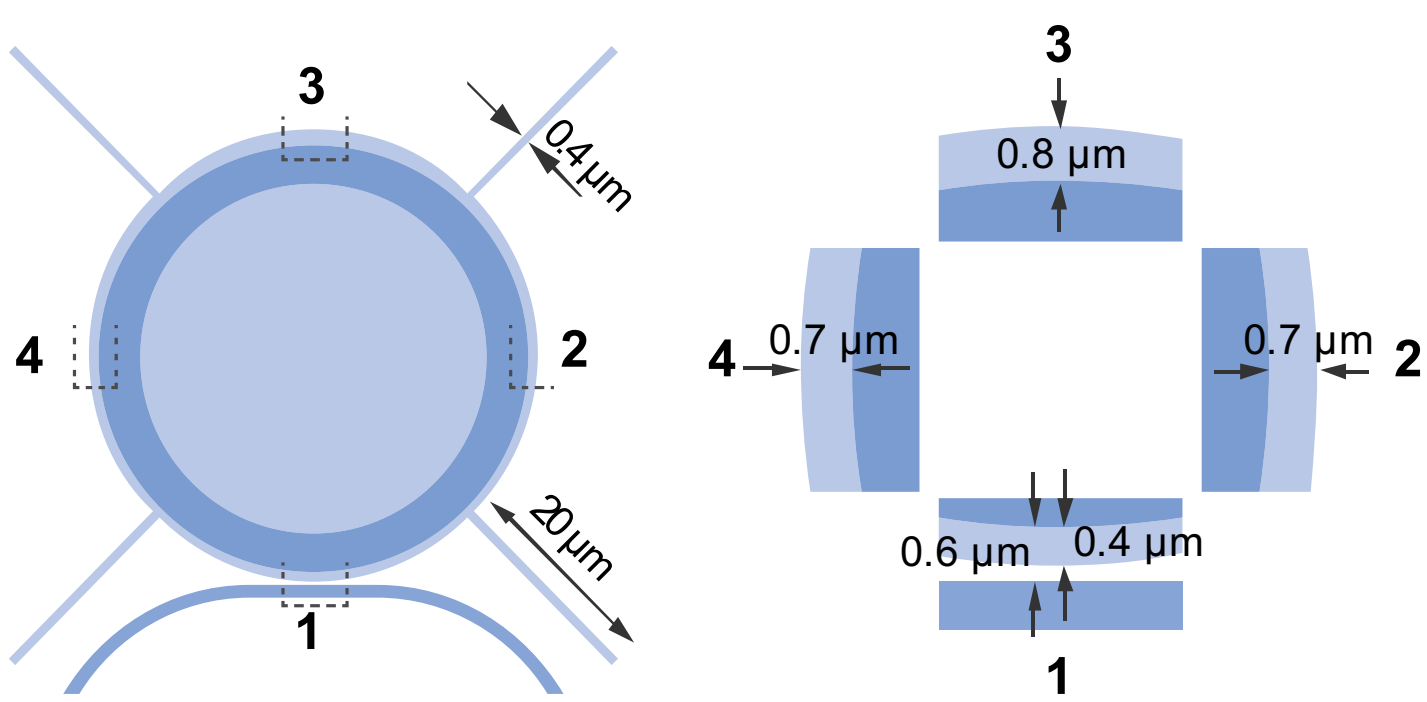

Fig. S1 | Structural design. a. Fabrication masks for the two-step etching. Dark blue and light blue indicate the first and second layers, respectively. **b.** Dimensions at points 1, 2, 3, and 4, as marked in **a.**

2. THERMO-OPTICAL (TO) EFFECT

Figure S2a plots the optical transmission spectrum near 1576 nm at different temperatures. As temperature increases, the resonance dip moves to a longer wavelength, indicating an increase in the effective refractive index. Figure S2b plots the resonance wavelengths as a function of temperature. A linear fit yields a TO coefficient of 3.83×10^{-5} K^{-1}. The input power was

maintained at a low level to suppress potential nonlinear effects. We also observe a distinct decrease in extinction ratio and increase in linewidth as temperature increases.

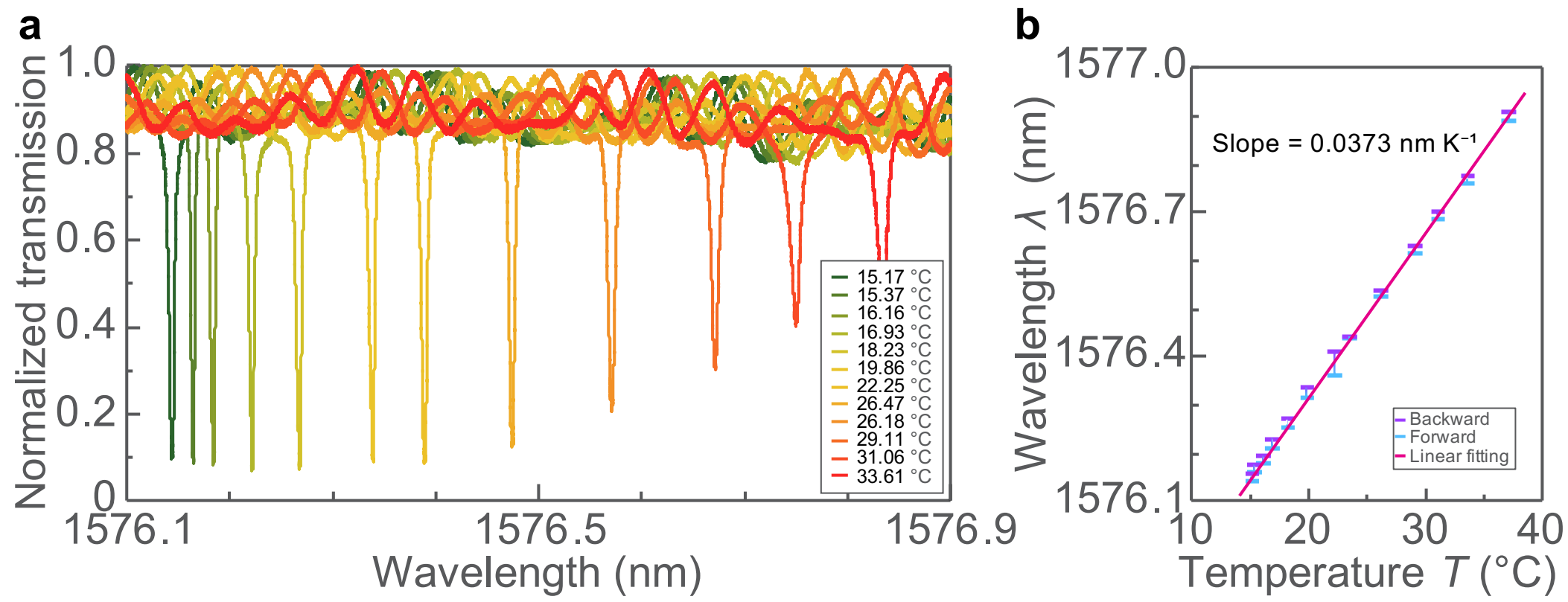

Fig. S2 | Thermo-optical measurement. a. Measured optical transmission as the temperature varies from 15 to 33 °C. **b.** Resonance wavelength as a function of temperature with backward and forward sweeping.

3. SIMULATED TCFS OF DIFFERENT MECHANICAL MODES

Figure S3a plots a higher frequency mechanical signal measured through intensity detection in Fig. 3d. Figure S3b plots the simulated temperature coefficients of frequency (TCFs) of four modes, where the target mode near 82 MHz exhibits the largest TCF, consistent with the experimental data.

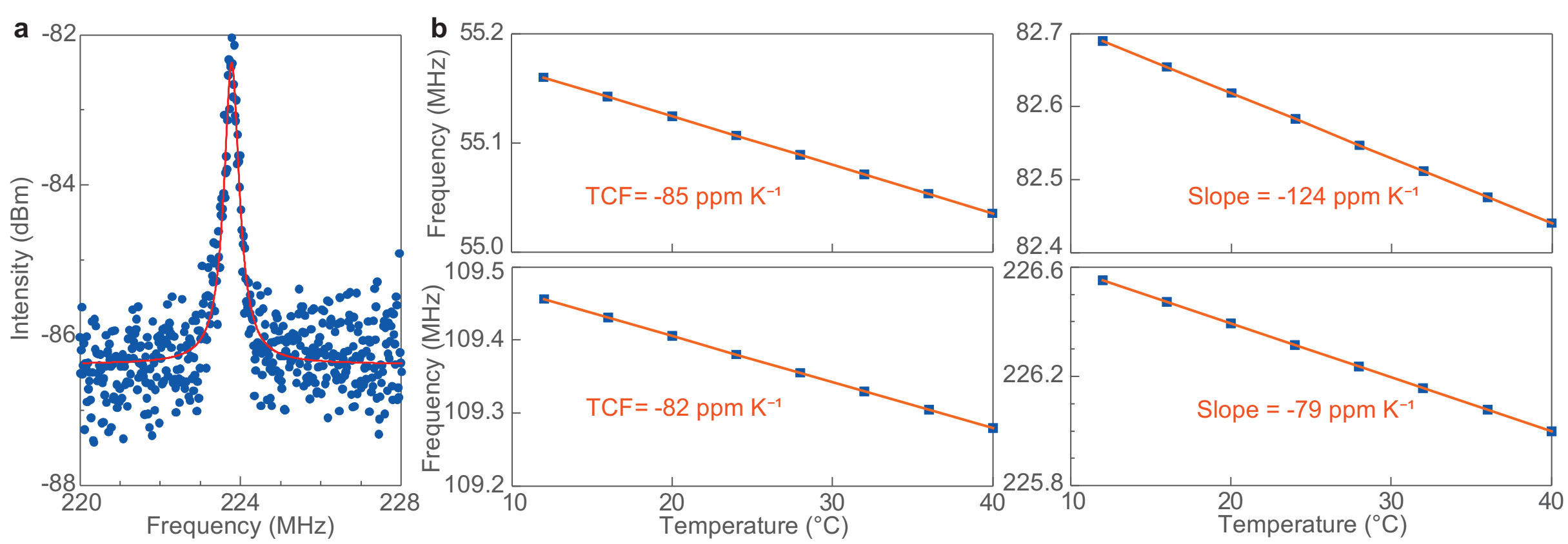

Fig. S3 | TCFs. a. Measured mechanical signal near 224 MHz. **b.** Simulated TCFs of four mechanical modes.

4. THERMAL CONDUCTANCE AND CAPACITANCE

In suspended optomechanical resonators, heat dissipates through three main pathways: anchor conduction (G_{anchor}), air convection (G_{air}), and surface radiation (G_{rad}). In the ambient environment, G_{air} dominates the total thermal conductance and is primarily governed by device geometry and surface area. In this limit, the intrinsic thermal time constant simplifies to $\tau = C_{th}/G_{th} \approx C_{th}/G_{air}$, and the temperature response to a step heat input follows a single exponential:

$$T = T_0 + \Delta T \cdot (1 - e^{-\frac{t}{\tau}})$$

However, when the heat source is located away from the resonator, as in our experimental configuration, an additional spreading thermal resistance is introduced between the heat source and the resonator body (Fig. S4a). This creates a second thermal pole, producing a sigmoid-shaped temperature response that a single exponential cannot capture. In this case, the response is more accurately described by a two-pole model:

$$T = T_0 + \Delta T \cdot (1 - \frac{\tau_1 e^{-\frac{t}{\tau_2}} - \tau_2 e^{-\frac{t}{\tau_1}}}{\tau_1 - \tau_2})$$

where τ_1 is the fast time constant associated with internal thermalization of the resonator body, and τ_2 is the slow time constant governing heat dissipation through the dominant thermal pathway. The measured response time of our device is characterized by τ_2.

To verify this, we simulate three configurations (Fig. S4b) using the Heat Transfer Module in COMSOL Multiphysics:

i. A 10 μm heat source placed at the center of the resonator, monitor temperature at points a and b.

ii. A uniform heat source distributed over the entire resonator, monitor temperature at points b.

iii. A heat source applied to the top surface of the silicon substrate, monitor temperature at point b.

Figures S4c and S4d show the simulated time-domain responses fitted with a two-pole model for each configuration. In configuration (i), point b exhibits a significantly slower thermal response (τ = 5.8 μs) compared to point a (τ = 2.4 μs), reflecting the additional spreading thermal resistance between the center heat source and the rim. In configuration (ii), a uniformly distributed on-chip heat source yields a response time of τ = 5 μs at point b, which represents the intrinsic thermal time constant. In configuration (iii), locating the heat source on the silicon substrate further slows the response to τ = 6.8 μs due tothe additional thermal resistance of the substrate-to-resonator interface.

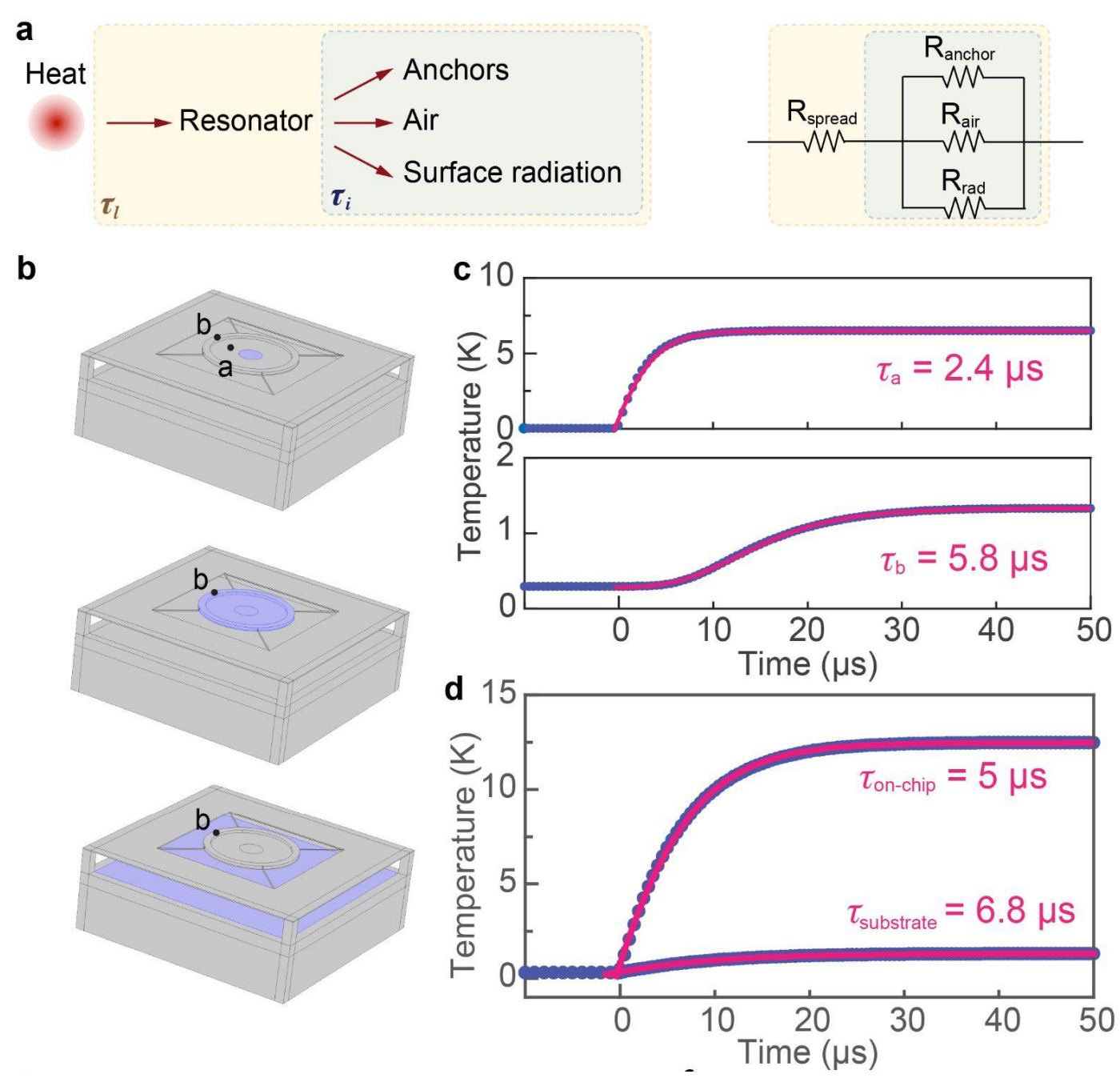

Fig. S4 | **a**. Heat dissipation model in our suspended optomechanical system. **b**. Three simulation configurations constructed in COMSOL Multiphysics, where the blue regions denote the heat sources. **c**, **d**. Simulated time-domain responses with two-pole fits for the three configurations.

5. FREQUENCY NOISE, ALLAN DEVIATION, AND NOISE EQUIVALENT POWER (NEP)

Frequency noise is commonly quantified by the power spectral density (PSD) of frequency fluctuations $S_y(f)$, which is particularly important in optomechanical sensing because it determines the minimum detectable frequency shift. Depending on the underlying noise process, the PSD scales with Fourier frequency f as $S_y(f) \sim f^{\alpha}$, where α = −2, −1, 0, 1, or 2 [1]. However, the PSD does not directly reveal how stability evolves with measurement time τ. The temporal frequency stability of a mechanical resonance is therefore quantified using the Allan deviation σ_y

$$\sigma_y^2(\tau) = 2\int_0^{\infty} S_y(f)\frac{\sin^4(\pi f\tau)}{(\pi f\tau)^2}df \text{ (S1)}$$

Accordingly, σ_y follows a power law in τ as $\sigma_y(\tau) \propto \tau^{\mu}$, where $\mu = -\frac{\alpha+1}{2}$. Here, we consider τ within the range of 10^{-4} s to 100 s. The standard five exponents (f^2, f^1, f^0, f^{-1}, f^{-2}) are usually sufficient to describe most practical frequency noise sources in oscillators, which together result in an overall Allan deviation

$$\sigma_y(\tau) = \sqrt{\sum_{\alpha=-2}^{2}\sigma_{y,\alpha}^2(\tau)} \text{ (S2)}$$

Among all noise sources, thermal-fluctuation-induced frequency noise establishes a fundamental limit to the frequency stability of the mechanical resonator. We therefore examine how thermal fluctuations contribute to frequency noise and analyze the corresponding scaling of σ_y with respect to τ. The first contribution is thermodynamic noise, for which the temperature fluctuation $S_T(f)$ directly modulates the mechanical frequency, as

$$S_f(f) = \left(\frac{df_m}{dT}\right)^2 S_T(f) \text{ (S3)}$$

and $S_T(f)$ is typically Lorentzian as

$$S_T(f) = \frac{4k_B T^2 G_{th}}{(2\pi f C)^2 + G_{th}^2} \text{ (S4)}$$

where k_B and T denote the Boltzmann constant and absolute temperature, respectively. When $f \gg \frac{G_{th}}{2\pi C}$, $S_f(f) \propto f^0$ and $\sigma_y(\tau) \propto \tau^{-\frac{1}{2}}$, it behaves as white frequency noise; when $f \ll \frac{G_{th}}{2\pi C}$, $S_f(f) \propto f^{-2}$ and $\sigma_y(\tau) \propto \tau^{\frac{1}{2}}$, it behaves as random-walk frequency noise. The second contribution is

thermomechanical noise, which arises from thermal forces $S_F(f)$ acting on the mechanical mode, as

$$S_f(f) = \left(\frac{g_{OM}}{2\pi}\right)^2 \frac{S_F(f)}{m_{eff}^2\left[\left(\omega_m^2-(2\pi f)^2\right)^2+(2\pi f \Gamma_m)^2\right]} \quad \text{(S5)}$$

where $S_F(f) = 4k_B T m_{eff} \Gamma_m$. m_{eff}, Γ_m, and g_{OM} are the effective mass, mechanical damping rate, and optomechanical coupling rate. When $f \ll f_m$, $S_F(f) \approx$ const and $\sigma_y(\tau) \propto \tau^{\frac{1}{2}}$, which behaves as white frequency noise; when $f \gg f_m$, $S_F(f) \approx f^{-4}$ and $\sigma_y(\tau) \propto \tau^{\frac{3}{2}}$, Using these equations, we can fit the measured σ_y before and after oscillation, as shown in Fig. S5. By combining σ_y with the device G_{th} and TCF, we can convert measured frequency fluctuations into noise-equivalent power (NEP), which is a fundamental figure of merit for sensors and is defined as

$$NEP_T = \frac{\sqrt{\tau}\sigma_f(\tau)G_{th}}{TCF} \quad \text{(S6)}$$

Optimizing the resonator geometry can further reduce the NEP and improve device performance. During operation in air, $G_{th,air}$ dominates the thermal conductance. The thermal-fluctuation noise NEP_T and thermomechanical displacement noise NEP_{thermo} can therefore be expressed using Eq. (2) of the main text:

$$NEP_T \propto \sqrt{\frac{R^4}{w_r^2 t^2 d^3}}, NEP_{thermo} \propto \sqrt{\frac{R^3}{w_r t d^2}} \quad \text{(S7)}$$

In this case, decreasing R and increasing d can substantially reduce both NEP_T and NEP_{thermo} in air. Increasing w_r and t provides additional noise suppression, although it may increase optical and mechanical losses. During operation in vacuum, $G_{th,air}$ is set to zero, leaving only the anchor and radiation terms. Because $G_{th,anchor}$ and $G_{th,rad}$ are comparable, the dominant contribution depends on the specific structural design.

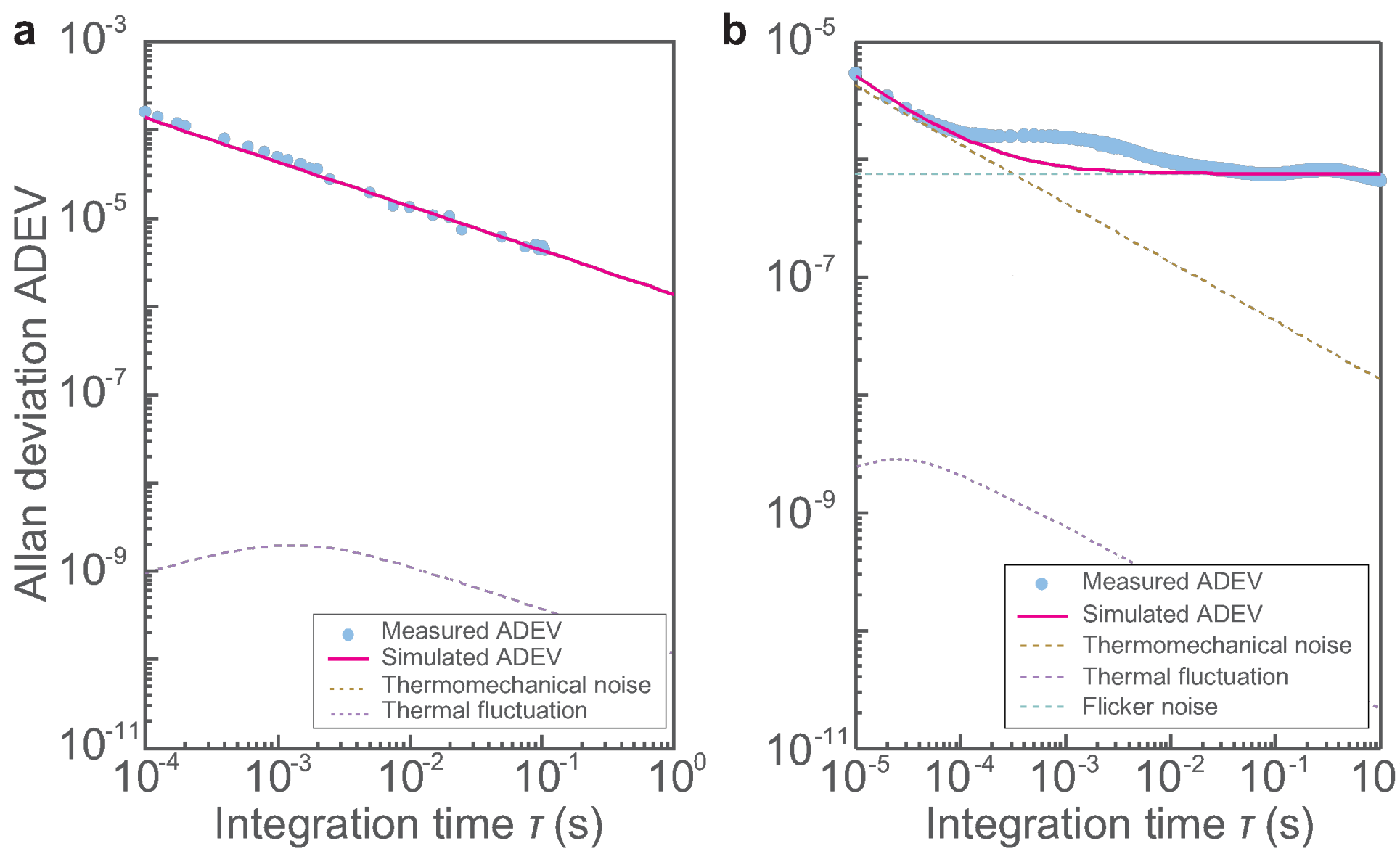

Fig. S5 | Allan deviation analysis. a. Reconstructed σ_y from oscilloscope measurement before oscillation with the fitting curves. **b.** Measured σ_y from the frequency counter after oscillation with the fitting curves.

Table S1 | Comparison of circular-shaped optomechanical resonators in air

Structure	Material	Supporting architecture	Diameter (µm)	f_m (MHz)	Q_m	$f_m \cdot Q_m$ (Hz)	Q_o	g_{OM} (kHz)
Microdisk	Si [2]	P	16	316	2141	6.77×10^{11}	1.03×10^{6}	NA
	Si [3]	P	4	1294	3300	4.27×10^{12}	3.5×10^{5}	108
	4H-SiC [4]	P	9	950.1	12000	1.14×10^{13}	1.2×10^{6}	NA
	LN [5]	P	12	375.9	5360	2.01×10^{12}	2.25×10^{5}	11.6
	GaAs/AlGaAs [6]	P	5	551.1	1300	7.16×10^{11}	2×10^{5}	NA
	3C-SiC [7]	P	4	1690	5500	9.3×10^{12}	3.8×10^{4}	29
	GaP [8]	P	8	488	640	3.12×10^{11}	2.8×10^{5}	30
Microtoroid	Silica [9]	P	72	4.4	3500	1.54×10^{10}	$>1\times10^{7}$	41.8
Microring/ membrane	SiN/SiO_2 [10]	membrane	470	0.29	29	8.41×10^{6}	1.33×10^{6}	NA
Microring	Si [11]	A	41.6	57	1000	5.7×10^{10}	2.3×10^{5}	NA
	Phosphosilicate Glass [12]	A	105	18.6	1050	1.95×10^{10}	2.8×10^{6}	NA
	AlN [13]	A	75.2	47.3	1742	8.24×10^{10}	1.25×10^{5}	NA
**This work**	**LN**	A	**40**	**83.7**	**1117**	$\mathbf{9.4\times10^{10}}$	$\mathbf{1\times10^{6}}$	**4.2**

P, central-pedestal supported; A, anchor supported; NA, data not provided.

References

[1] Riley, W. J. NIST Special Publication 1065: Handbook of frequency stability analysis (US Government Printing Office, Washington, DC, USA, 2008).

[2] Hermouet, M. et al. Proceedings 1, 347 (2017).

[3] Jiang, W., Lu, X., Zhang, J. & Lin, Q. Opt. Express 20, 15991–15996 (2012).

[4] Liu, Y., Sun, W., Abiri, H., Feng, P. & Li, Q. Photon. Res. 13, 2531–2538 (2025).

[5] Jiang, W. & Lin, Q. Sci. Rep. 6, 36920 (2016).

[6] Sentre-Arribas, E. et al. ACS Sens. 9, 371–378 (2024).

[7] Lu, X., Lee, J. & Lin, Q. Sci. Rep. 5, 17005 (2015).

[8] Mitchell, M., Hryciw, A. C. & Barclay, P. E. Appl. Phys. Lett. 104, 141104 (2014).

[9] Kippenberg, T. J., Rokhsari, H., Carmon, T., Scherer, A. & Vahala, K. J. Phys. Rev. Lett. 95, 033901 (2005).

[10] Cao, X. et al. Light Sci. Appl. 15, 171 (2026).

[11] Yu, Y., Xi, X. & Sun, X. Light Sci. Appl. 11, 328 (2022).

[12] Chen, I., Li, B., Lee, S., Chakravarthi, S., Fu, K. & Li, M. Nat. Commun. 14, 7594 (2023).

[13] Xiong, C., Sun, X., Fong, K. Y. & Tang, H. X. Appl. Phys. Lett. 100, 171111 (2012).
